# Smartphone applications supporting self-management programme for adults with Chronic Obstructive Pulmonary Disease: A Scoping Review

**DOI:** 10.1371/journal.pdig.0000532

**Published:** 2024-06-13

**Authors:** Lisa Glynn, Margaret Mc Cann, Catherine Mc Cabe

**Affiliations:** 1 School of Nursing and Midwifery, University of Galway, Ireland; 2 School of Nursing and Midwifery, Trinity College, Dublin 2, Ireland; Brown University, UNITED STATES

## Abstract

Introduction: Chronic Obstructive Pulmonary Disease (COPD) significantly impacts on both the quality and quantity of life for patients due to frequent exacerbations requiring hospital admissions resulting in increased morbidity and mortality. A self-management programme purpose is to increase one’s knowledge, confidence, and skills to self-manage their chronic illness such as COPD. Objective: The objective of this review will therefore answer the following research question: *What is the current literature pertaining to the use of a smartphone app in supporting a comprehensive self-management programme among COPD patients*? A preliminary search was conducted in, Medline, Embase and CINAHL databases to ascertain index terms and keywords. Following this a rigorous search was carried out on Medline, Embase, CINAHL, Web of Science and ASSIA. The findings from this search are presented in tabular form using the PRSIMA flow diagram. Results: In this review, fifteen studies met the inclusion criteria. Across all studies participants engaged with the app and developed self-management skills and knowledge to manage their chronic illness. However, engagement with the app without third party involvement declined over time. Technical issues did not cause harm to participants but in some cases contributed to reduced engagement. Smartphone self-management apps empowered a cohort of COPD participants to engage in managing their chronic illness which proved useful in detecting exacerbations earlier resulting in reducing the need for hospitalisations over a three-to-six-month period. By reducing hospitalisations incurred a cost savings for health care and an improved quality and quantity of life for these participants. Conclusion: It is evident from the literature that smartphone self-management apps may positively influence participants self-management decisions in terms of knowledge, increase physical activity, self-efficacy that may result in reduced hospitalisation and improved quality of life. It is clear that technical issues and sustained engagement over longer periods of time remains a challenge.

## Author summary

This scoping review provides an overview on the current literature relating to smartphone apps in supporting a comprehensive self-management programme for adults with COPD. Continued engagement with smartphone apps over longer periods of time remain a challenge and this requires further attention to ensure continued engagement. There was some benefit shown in using smartphone apps to support a comprehensive self-management programme however, further research is required with larger sample sizes over a 12-month period in order to determine the effect of smartphone apps supporting a comprehensive self-management programme for adults with COPD.

## Introduction

Chronic Obstructive Pulmonary Disease (COPD), is an incurable chronic lung disease largely caused from extensive exposure to cigarette smoking and other noxious gases. The development of COPD is further influenced by host factors such as genetics, childhood abnormal lung development, air pollution and aging [1]. It results in persistent respiratory symptoms, such as a chronic productive cough, breathlessness, wheeze, and airflow limitation [1]. After ischaemic heart disease and stroke, COPD is the third leading cause of all deaths worldwide (6%) [2].

COPD exacerbations are defined as an acute event described by a worsening of the patient’s respiratory symptoms that is beyond normal day-to-day variations [1]. Regular exacerbations with ongoing, progressive lung function decline because of COPD can significantly impact the quality and quantity of life resulting in increased morbidity and mortality [1,3,4,5].

COPD exacerbations are the most frequent presentations to the General Practitioner (GP) and hospital setting among this cohort worldwide resulting in increased morbidity and mortality among this cohort [1,3]. To better manage these exacerbations, there is a need to encourage these patients to engage in a self-management programme to self-manage their disease resulting in earlier recognition of signs and symptoms of an exacerbation, prompting earlier treatment thereby reducing hospitalisations among this cohort [1,6,7].

A self-management programme purpose is to increase one’s knowledge, confidence, and skills to self-manage their chronic illness [1,8]. By engaging in a self-management programme results in lifestyle and behavioural changes to improve social, emotional, and physical aspects of their life resulting in managing their chronic illness. This results in improved outcomes for patients, including enhancing quality of life, empowerment to self-manage their chronic illness resulting in reduced healthcare hospitalisation, morbidities, and premature death [1,8,9,10,11,12,13,14].

Since the COVID-19 pandemic there has been a rapid evolution on the use of smartphones, in the delivery of healthcare [15,16]. The COVID-19 pandemic has positively influenced the older population view on technology resulting in the increased use of smartphone apps among this cohort prior to the pandemic [16]. The smartphone has many benefits such as providing convenience communication with the patient and healthcare professional, offers portability, Bluetooth and internet connection allowing for the use of various smartphone apps to work anywhere at any time. Also, smartphones support behaviour changes by providing education, interactive feedback, motivational messages, and access to online resources available at any time and are generally available at a low cost in comparison to other digital technologies such as a computer or tablet. Previous studies utilising self-management programmes via a smartphone app have illustrated some positive clinical health outcomes that prevented deteriorating health conditions necessitating a hospital admission across several chronic illnesses such as diabetes, cardiac, asthma and COPD [6,7,17,18,19]. However, smartphone technology investigated in past studies among COPD patients mainly focused on one of the following, physical activity, pulmonary rehabilitation, education, recording of symptoms or using medical devices to record physiological parameters [20,21,22]. A self-management programme requires a comprehensive view to capture the entire purpose of a self-management programme resulting in increasing one’s knowledge, confidence, and skills to self-manage their chronic illness [1,8]. A comprehensive self-management programme includes two or more of the following, education, breathlessness, physical activity, pulmonary rehabilitation, medication adherence, recording of symptoms and using medical devices to record physiological parameters. It is expected that smartphone apps may support a comprehensive self-management programme thereby improving clinical health outcomes resulting in reduced hospitalisations for patients with COPD. However, there is no conclusive scientific evidence available supporting the use of smartphone apps delivering a comprehensive self-management programme that improves clinical health outcomes for patients with COPD. Therefore, there is a strong need for more research surrounding smartphone apps supporting comprehensive self-management programme among COPD patients to better understand its role in healthcare. The COVID-19 pandemic has contributed to further accelerate the work on smartphones apps supporting a self-management programme to support vulnerable patients such as those with COPD in their home [23,24,25]. Given this, recent studies have been conducted within this domain, exploring smartphones apps supporting a comprehensive self-management programme for people with COPD.

This scoping review will build on existing reviews and will help to supplement and understand the existing literature on smartphone apps supporting a comprehensive self-management programme among COPD patients. The objective of this review will therefore answer the following research question: *What is the current literature pertaining to the use of a smartphone app in supporting a comprehensive self-management programme among COPD patients*?

## Review questions

1. *What is the current literature pertaining the use of a smartphone app in supporting a comprehensive self-management programme among COPD patients*?
2. *Have these apps been evaluated in terms of clinical health outcomes and if so*, *what are the outcomes of these evaluations*?

## Inclusion criteria

The inclusion criteria were designed according to the Johanna Briggs Institute (JBI) (2020) manual of scoping review methodology, comprising of Population, Concept, Context and Study Design [26], and are defined in Table 1.

**Table 1:** Inclusion Criteria.

**Criteria**	**Description**
**Population**	The scoping review will include studies that recruited COPD patients irrespective of severity of disease, ethnic background, geographical area living at home using a smartphone app self-management programme.
**Concept**	To explore the literature on the use of a smartphone app supporting a comprehensive self-management programme and how these apps have been evaluated in terms of clinical health outcomes. Smartphone apps will be defined as software that provides a particular function incorporated into smartphones to improve an health outcome, research, and health care services [22]. A comprehensive self-management programme will include two or more of the following: education, physical activity, breathlessness, recording of exacerbations, medication adherence, pulmonary rehabilitation, recording of symptoms or using medical devices to record physiological parameters. This review will not include apps on technologies such as personal computers, iPad, Android tablets, Zoom, and Skype. Also, studies that included telemonitoring or clinic follow up visits.
**Context**	The context will include hospital, primary care and community settings who care for COPD patients. COPD patients living in long-term care facility will be excluded.
**Study Design**	Study design such as editorials, quantitative research designs, qualitative research designs, mixed methods and grey literature with relevant data will be included.
**Language**	No restrictions on languages.
**Year**	No restrictions based on the year of publication.
**Publication Status**	No restrictions will be employed based on publication status.

Table 1 provides an outline of the inclusion criteria used for this scoping review in terms of population, concept, context, study design, language, year, and publication status.

## Types of sources

To answer the research question, this scoping review will explore the literature using quantitative, qualitative and mixed methods design. Also, editorials and grey literature with relevant data will be included in this review.

## Methods

This scoping review is aligned with the JBI methodology framework for scoping reviews and the Preferred Reporting Items for Systematic Reviews and Meta-Analysis Scoping Review (PRISMA-ScR) extension checklist was used (S1 Appendix) [27]. This review was conducted in accordance with an *a* priori protocol published in Open Science Framework on the 31-05-2022.

### Search strategy

A preliminary search was conducted in, Medline, Embase and CINAHL databases to ascertain index terms and keywords. Following this a rigorous search will be carried out using the index terms and keywords on the following databases: Medline, Embase, CINAHL, Web of Science and ASSIA. These databases were chosen based on their relevance to the review questions. The search strategy was developed for each database, and with the aid of an experienced librarian a review of electronic search strategies was carried out (S2 Appendix). Hand searching of the literature occurred from the references of included articles. Grey literature was also be searched in appropriate organization sites (including on ProQuest Dissertations and Theses, RIAN, LENUS, Health Service Executive (HSE), Agency for Healthcare Research and Quality, Health Information and Quality Authority, Health Standards, and National Institute for Health and Care Excellence).

Studies that do not meet the inclusion criteria were excluded.

### Study selection

The results from the search were imported into Endnote v.X9 and duplicates were removed. Following this, citations were imported into Covidence, and the results of the search were reviewed. All studies were examined. Screening was completed at both the title/abstract level and full-text level. A third person would have been consulted if uncertainties remained between reviewers. A PRISMA flow diagram illustrates the results from the screening process with explanations for full text exclusions. The flow chart clearly details the review decision process, indicate the results from the search, removal of duplicate citations, study selection, full retrieval, and additions from reference list searching and final summary presentation. Inter-rater agreement was calculated due to the iterative nature of the review.

### Data extraction

A data extraction form was used to streamline data collection and to ensure that all relevant data was gathered (S3 Appendix). Two independently reviewers piloted the extraction forms on a random sample of five articles. One reviewer charted the data, and the second reviewer verified the extracted data. A third person would be consulted to achieve consensus on coding, if consensus between the two independent reviewers can not be reached, and the data extraction form would be adjusted if required. Authors of papers were contacted to request missing or additional data for clarification, where required. A draft charting table were developed to record characteristics of the included studies and the key information relevant to the review objective (Table 2). Given the iterative nature of scoping reviews, additional information was collected as deemed appropriate.

## Results

### Study inclusion

**Fig 1:**
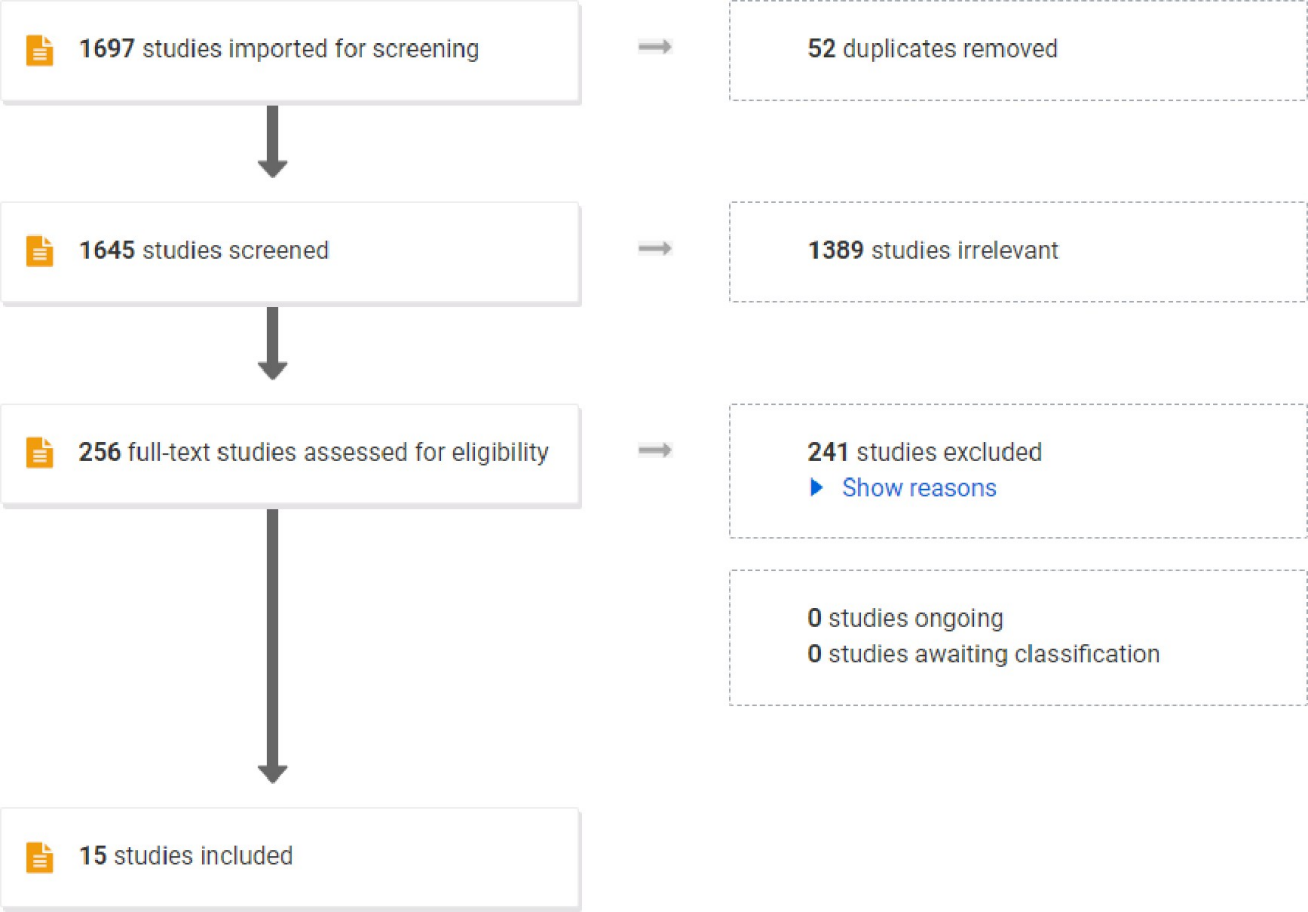
PRISMA Flow Chart represents the screening process for this scoping review. It highlights the number of studies assessed and the number of studies were excluded as they did not meet the eligibility criteria. Studies were excluded due to wrong intervention, third party engagement (telemonitoring and follow up clinic visits), wrong study design, wrong population, and non-English language studies. This PRISMA Flow Chart illustrates the number of studies included in this scoping review.

During the initial phase, 1,697 studies were included, 52 were duplicates and 1,645 studies were screened. Following this, 1,389 studies did not meet the review inclusion criteria and were discarded. In the full text review 256 studies were reviewed and 15 studies met the inclusion criteria (Fig 1). The main reasons for exclusion were as follows, wrong intervention (tablet, iPad or personal computer), third party engagement (telemonitoring and follow up clinic visits), wrong study design and incorrect population.

### Characteristics of included studies

Study characteristics are reported in Table 2. All 15 studies were published after (2019) with the majority published in (2021). The studies were conducted in several countries including United States [28,29,30,31] Spain [32,33,34], Netherlands [35,36], United Kingdom [37,38], China [39], Germany [40], Switzerland [40], Korea [11], and Wales [41]. Eight studies [11,29,30,31,35,36,37,41] included a population less than 50 participants. The largest number of participants in a study included in this review was 184 participants [28]. Participants were generally aged greater than 60 years of age and the proportion of males were higher in comparison to females in some studies [11,32,33,36,37,38,39,41]. Study duration varied from 3 weeks to 12 months [36,39]. Three studies [30,31,33] used devices to measure physiological parameters such as a spirometer to measure lung function, pulse oximeter to measure oxygen saturations and a muscle strength training device to measure inspiratory and expiratory lung strength. Six studies included all severity groups of COPD [28,29,31,37,39,41]. The self-management app was provided by the researcher to the participants once they agreed to partake in the study.

**Table 2:** Study Characteristics.

Author & Year	Country of Origin	Setting	Severity of COPD Included (A,B,C,D)	Study Design	Sample Size	Average Age	Sex M %	Duration	Primary Outcome	Other Outcomes
Bowler *et al*. (2019)	USA	Community	All	Observational study	184	66	45%	3 weeks	Exacerbations	• Physical Activity • Inhaler usage • Respiratory symptoms • PROactive Scale
Alharbey *et al*. (2019)	USA	Community	All	Mixed Method	21	73.4	48%	1 month	Knowledge and awareness regarding COPD	• Self-efficacy • Engagement • Quality of life • Recording oxygen saturations • Education • Perceived Seriousness • Patient Empowerment
North *et al*. (2020)	United Kingdom	Community and hospital	All	Parallel arm feasibility RCT	41	66.6	59%	90 days	Recovery rate of symptoms	• Exacerbations • Hospitalisations • Quality of life • Inhaler technique Engagement • Anxiety & Depression
Park *et al*. (2020)	Korea	Community	A, B & C	RCT	42	67.88	78.6%	6 months	Self-efficacy	• Hospitalisations • Engagement • Education • Physical Activity • Quality of life
Hermosa *et al*. (2020)	Spain	Community	B,C,D	Prospective cohort Study	116	66.51	78,4%	6 months	Frequency and characteristics of an exacerbation	• Hospitalisations • Engagement • Self-efficacy
Crooks *et al*. (2020)	United Kingdom	Community	A&B	RCT	60	66	64.5%	3 months	CAT at 90 days and inhaler errors	• App usage • Patient Activation Measurement • Medication use. • Quality of life • Exacerbation
Wang *et al*. (2020)	China	Community	All included	RCT	68	63.9	81%	12 months	Health Related Quality of Life	• Self-management behaviour • Self-efficacy • Physical Activity • Disease Education
Hermosa *et al*. (2021)	Spain	Community	C & D	Prospective cohort	69	66.84	82.6%	6 months	Exacerbations detected through the app	• Lung function • Breathlessness • Recording of symptoms • Medication use • Disease education • Engagement
Miller *et al*. (2021)	USA	Community	B & C	RCT	30	58.4	36.6%	6 weeks	Engagement & acceptability	• Physical Activity • Recording of symptoms • Breathlessness • Medication adherence • Respiratory muscle strength training
Knox *et al*. (2021)	Wales	Community	All	Quantitative, single-arm, clinical pilot study	25	64	76%	6 weeks	Engagement, safety, and efficacy	• Knowledge & confidence • Hospitalisations • Quality of life • Breathlessness
Kooij *et al*. (2021)	Netherlands	Community	B,C,D	Mixed Methods	39	62.2	23%	8 weeks	Engagement	• Patient Satisfaction • Education • Medication Adherence • Expectations of the app • Hospitalisations
Batlle et al. (2021)	Spain	Community	C&D	RCT	87	82	50%	3months	Use of health care service	• Engagement • Physical Activity • Quality of life • Patient empowerment • Cost effectiveness • Recording of oxygen saturation
VanBuul *et al*. (2021)	Netherlands	Community	B,C,D	Retrospective study with a pre-post research design	29	67.4	55.1%	12 months before 18 months after using the app	Hospitalisations	• Detection of exacerbations • Symptom Recorder • Monitor steps • Disease education
Spielmanns et al. (2022)	Germany and Switzerland	Community	B,C,D	RCT	60	64.8	39%	6 months	Physical Activity	• Engagement • Quality of life • Anxiety & Depression • Recording of symptoms
Gelbman et al. (2022)	USA	Community	All included	Prospective observational pilot study	19	79.6	47.4%	8 weeks	Patient engagement and satisfaction	• Lung function • Oxygen Saturation • Symptoms recorded • Medication use • Nebulizer use • Engagement

Study Characteristics provides an overview of the studies included in this review in terms of author, year, country of origin, setting, severity of COPD, study design, sample size, average age of participants, sex, duration of the study, primary outcomes and secondary outcomes.

### Primary outcome

Five studies primary outcome focused on engagement [30,31,35,38,41] while three of these studies also included acceptability [30,32] patient satisfaction [31], safety and efficacy [41]. All studies varied in study design with all studies including less than 50 participants. Two studies reported continued engagement throughout the designated timeframe, 6 [30] and 8 [31] weeks respectively. Knox *et al*. (2021) reported participants engaged with the app 31.8 days out of 42 days. Studies [33,35,37,38,40,41] reported high levels of engagement at the beginning of the study however engagement declined over time. COPD participants engaged with the app despite age, gender, or severity of disease [31]. Participants found the app relatively easy to use resulting in being positively satisfied and incurred no safety issues using the app, no adverse events occurred [30,31,35,40,41]. The app was accepted by the majority of participants who reported they would use the app for longer durations [30,32,35]. However, technological problems were noted in all studies and in some cases affected engagement levels [30,31].

The remaining eleven studies reported overall good compliance using the app resulting in empowering participants to engage with self-care activities to manage their chronic illness. One of the studies [30] gave a financial reward throughout the study resulting in continued engagement from participants.

Four studies [28,32,33,36] identified self-management apps to be useful in detecting exacerbations and two studies highlights that self-management apps may improve exacerbation recovery rates thereby lowering re-exacerbation or readmission rates over 6 months [37,36] demonstrating a cost effectiveness in using self-management apps. Cost effectiveness was explored [34] on the implementation of a self-management mobile app which proved effective in reducing hospitalisations by 50% for each patient resulting in cost savings of more than $850 US dollars per patient. Furthermore, unplanned visits were reduced by 57% [34]. Also, the app proved useful in identifying the exact number of exacerbations in comparison to participants recall of their exacerbation history which may prove beneficial to clinicians during a clinical visit [33]. Only one of these studies [28] included all categories relating to severity of disease. Study designs varied from pre-post design to observational with no parallel control group [28,32,33,36].

### Other outcomes

#### Quality of life

Seven studies [11,29,34,37,38,40,41] reported on health-related quality of life (HRQoL), two studies report a statistically significant difference in terms of HRQoL between the intervention and control group [11,40]. Sustained self-care behaviour was observed over 6 [11] to 12 [39] months, demonstrating the feasibility and efficacy of the self-management app. These studies were not fully powered which may question the significance of the statistical analysis. One study [41] did not have a control group as a comparator.

#### Recording measurements

Five studies [11,28,30,34,36] recorded physical activity using accelerometers. One study [39] used a questionnaire to evaluate physical activity among participants. Studies [11,34,39] reported a significant increase in physical activity in comparison to a control group. Two studies [28,36] did not use a control group. Miller *et al*. (2021) reported technological issues recording physical activity via an accelerometer on a smartwatch synching with the mobile app.

One study [31] measured lung function using a spirometer and oxygen saturation via a pulse oximeter. Miller *et al*. (2021) measured inspiratory and expiratory lung muscle strength using a muscle strength training device.

#### Disease education

Six studies included disease education in the app [11,33,35,36,39,41]. Most participants reviewed the education section on the app resulting in improved self-management knowledge and behaviour [11,29,33,35,36,39]. This knowledge improved self-care of their chronic illness resulting in recognising exacerbations earlier resulting in participants self-managing the exacerbation or pursuing care earlier thereby avoiding hospitalisations [33,35,36,39]. One study [35] reported a statistically significant difference in knowledge and coping strategies for self-management care using the app.

## Discussion

COPD is an incurable chronic lung disease that results in frequent exacerbations resulting in hospitalisations thereby increasing mortality and morbidity among this cohort [1]. In recent times, due to the COVID-19 pandemic, studies have been conducted exploring smartphones apps supporting a comprehensive self-management programme for people with COPD. Interestingly, the COVID-19 pandemic has altered the way the older population view technology resulting in the increased use of smartphone apps in the delivery of healthcare [13,14]. With the increased use of smartphone apps within healthcare there is particular focus placed on data privacy. In (2018) Europe introduced the General Data Protection Regulation (GDPR) which was a significant piece of legislation to reinforce the principle of accountability and minimisation of personal data to be collected by healthcare organisations using smartphone apps [42]. However, with the increased use of smartphone apps and Artificial Intelligence (AI) there is a need to ensure stringent data sharing and privacy policies are in place to protect patients. Also, AI which may support smartphone app self-management programmes in terms of administrative tasks, tailoring individual self-management programme and updating apps with new educational content relating to COPD [43].

This scoping review illustrates that there are a lack of well-designed studies investigating smartphone apps in patients with COPD. This results in inconclusive evidence that smartphone apps supporting a comprehensive self-management programme has a statistically significant difference on clinical health outcomes in adults with COPD. This may be due to components of the self-management app across all fifteen studies were heterogeneous in nature, in terms of intervention, study design, including all categories of COPD participants and small sample size. Self-management apps via a smartphone only appeals to a certain cohort of patients for many reasons such as digital literacy, not owning a smartphone, lack of internet connectivity, reduction in dexterity to use a smartphone or simply not interested in self-managing their COPD [11,28,29,30,31,32,33,34,35,36,37,38,39,40,41,44].

Across all studies participants engaged with the app however, app usage likely declines over time [11,28,29,30,31,32,33,34,35,36,37,38,39,40,41]. Third party involvement may improve app engagement over time [33,35,38,41]. While technical issues did not cause harm but rather proved as an inconvenience to participants it also resulted in reduced engagement [30,31]. Technical issues included loss of functionality of the app, unable to pair devices with Bluetooth on the smartphone, duplicate recordings, updating and advancing the app during the study. To achieve sustained engagement with the app it was noted the app must be personalised, easy to use, not over burdensome, have good technical support [30,31,44] and include follow up phone calls from a healthcare professional [11,30,34]. Also, financial incentives given at various points throughout the study contribute to sustained engagement with the app. The studies included in this review failed to demonstrate evidence of sustained engagement over a longer duration of twelve months.

It is evident exacerbations can negatively affect quality and quantity of life due to frequent hospitalisations thereby increasing mortality and morbidity [1,2]. A previous meta-analysis investigating a smartphone intervention compared to usual care demonstrated an 80% reduction in the probability of having an exacerbation [20]. It is important to note that outcome measures such as exacerbation rates may be unreliable as, only six studies reported on exacerbations [28,30,32,33,34,36]. Also, study designs differed, and exacerbations were reported using a range of varied tools. However, four studies illustrated the smartphone self-management apps empowered a cohort of COPD participants to engage in managing their chronic illness which proved useful in detecting exacerbations earlier resulting in reducing the need for hospitalisations over a three-to-six-month period [34,36,37,39]. By reducing hospitalisations incurred a cost savings for health care and an improved quality and quantity of life for these patients [34]. No studies in this review reported a reduction in hospitalisations or a reduced re-exacerbation rate over a twelve-month period.

Participant satisfaction and expectation was high while using the app [30,31,34,35,36,40,41]. No studies reported any participant safety issues while using the app. Clinical health outcomes such as physical activity, quality of life, self-efficacy, smoking cessation [39], medication adherence improved while using the smartphone self-management app. However, only two studies [11,40] reported a statistically significant difference in terms of HRQoL between the intervention and control group over a six-month period. This may be related to the small sample sizes which 13 [11,29,30,31,33,35,34,36,37,38,39,40,41] out of the 15 studies had less than 100 participants. Using the app illustrated a positive effect on knowledge around self-care for a chronic illness such as COPD resulting in improved clinical health outcomes for participants with COPD [11,28,32,33,34,35,36,39]. The provision of disease education using a smartphone app was shown to positively influence self-management decisions among the COPD population. As most patients diagnosed with COPD are greater than 60 years of age involving friends and or family members in the self-management process can be helpful in improving self-efficacy and quality of life among this cohort [41].

This scoping review provides an overview of the current literature and has summarized the findings on the use of a smartphone app supporting a self-management programme for COPD patients. Also, this review has adhered to a standard, rigorous methodological and reporting framework such as the JBI (2020) PRISMA-ScR. Limitations of this review include the absence of a risk of bias evaluations on the studies included in this review.

### Conclusion

There is insufficient evidence to support a statistically significant difference pertaining to a comprehensive self-management programme via a smartphone app on clinical health outcomes in adults with COPD. However, it is evident from the literature that smartphone self-management apps may positively influence participants self-management decisions in terms of knowledge, increase physical activity, self-efficacy that may result in reduced hospitalisation and improved quality of life. Although, technical issues and app usage over time remains a challenge. There is a need for improved digital advances in apps for COPD patients and for these apps to be successful there is a need for investment in technical advances and support to be readily available for participants to support engagement levels. Given technology is advancing rapidly, it is envisioned that smartphone apps will become a natural complement within clinical practice. Therefore, there is a need for more research exploring smartphone apps delivering a comprehensive self-management programme among all categories of the COPD population over a longitudinal period of twelve months.

## Supporting information

S1 Appendix PRISMA-ScR Checklist. (PDF)

S2 Appendix Search Strategy. (PDF)

S3 Appendix Data Extraction Form. (PDF)
