# Supplementary material for: Smartphone applications supporting self-management programme for adults with Chronic Obstructive Pulmonary Disease: A Scoping Review
Source: PLOS Digit Health. 2024 Jun 13;3(6):e0000532. doi: 10.1371/journal.pdig.0000532 (PMC11175531; doi:10.1371/journal.pdig.0000532)
Supplement: S2 Appendix — (PDF) [file pdig.0000532.s002.pdf]

S2 Appendix. Search Strategy

Concept: Apps

Medline (EBSCO) (MH "Mobile Applications") OR (MH "Smartphone") OR (MH "Cell Phone")

CINAHL (EBSCO) (MH "Cellular Phone+") OR (MH "Smartphone") OR (MH "Mobile Applications")

Embase (Elsevier) ('mobile phone'/exp) OR ('smartphone'/exp) OR ('mobile application'/exp)

ASSIA (Proquest) Keyword search only

Web of Science: TOPIC keywords only

Keywords for ASSIA & Web of Science: (app OR apps OR application*) NEAR/3 (“mobile phone*” OR “smart phone*” OR phone OR apps OR “cell phone*” OR Smartphone*) OR "Google Play" OR Istore OR "app store*"

Keywords for EBSCO databases (app OR apps OR application*) N3 (“mobile phone*” OR “smart phone*” OR phone OR apps OR “cell phone*” OR mobile* OR Smartphone*) OR "Google Play" OR Istore OR "app store*"

Keywords for Embase (app OR apps OR application*) N3 (‘mobile phone*’ OR ‘smart phone*’ OR phone OR apps OR ‘cell phone*’ OR mobile* OR Smartphone*) OR ‘Google Play’ OR Istore OR ‘app store*’

Concept 2: copd patients

Medline (EBSCO) (MH "Pulmonary Disease, Chronic Obstructive+") OR (MH "Pulmonary Emphysema") OR (MH "Bronchitis, Chronic") OR (MH "Asthma-Chronic Obstructive Pulmonary Disease Overlap Syndrome") OR (MH "Lung Diseases, Obstructive")

CINAHL (EBSCO) (MH "Pulmonary Disease, Chronic Obstructive+") OR (MH "Asthma-Chronic Obstructive Pulmonary Disease Overlap Syndrome") OR (MH "Lung Diseases, Obstructive+") OR (MH "Bronchitis, Chronic") OR (MH "Emphysema+")

Embase (Elsevier) ('chronic obstructive lung disease'/exp) OR ('lung emphysema'/exp) OR ('chronic bronchitis'/exp) OR ('asthma-chronic obstructive pulmonary disease overlap syndrome'/exp) OR ('obstructive airway disease'/exp)

ASSIA (Proquest) keyword search only

Web of Science: TOPIC keywords only

EBSCO, ASSIA & Web of Science Keywords: “Chronic Obstructive Pulmonary Disease*” OR “pulmonary Emphys*” OR “chronic bronchitis*” OR “Asthma-Chronic Obstructive Pulmonary Disease*” OR “chronic obstructive airway disease*” OR “chronic obstructive lung disease*” OR “obstructive Pulmonary disease*” OR “obstructive airway disease*” OR “obstructive lung disease*” OR COPD

EMBASE Keywords ‘Chronic Obstructive Pulmonary Disease*’ OR ‘pulmonary Emphys*’ OR ‘chronic bronchitis*’ OR ‘Asthma-Chronic Obstructive Pulmonary Disease*’ OR ‘chronic obstructive airway disease*’ OR ‘chronic obstructive lung disease*’ OR ‘obstructive Pulmonary disease*’ OR obstructive ‘airway disease*’ OR ‘obstructive lung disease*’ OR COPD

Proquest Dissertations and Thesis
