# Supplementary material for: Smartphone applications supporting self-management programme for adults with Chronic Obstructive Pulmonary Disease: A Scoping Review
Source: PLOS Digit Health. 2024 Jun 13;3(6):e0000532. doi: 10.1371/journal.pdig.0000532 (PMC11175531; doi:10.1371/journal.pdig.0000532)
Supplement: S3 Appendix — (PDF) [file pdig.0000532.s003.pdf]

S3 Appendix. Data Extraction Form

Review question: *What is the current literature pertaining the use of a smartphone app in supporting a self-management programme among COPD patients?*

**Name of Person extracting data**<br><br>**L. Glynn**	**Date of date extraction (dd/mm/yyyy):**

**Study ID** *(surname of first author and year first full report of study was published e.g. Smith 2001)*

**Report IDs of other reports of this study** *(e.g. duplicate publications, follow-up studies)*

**Notes:**

General Information

**Report author contact details (email address)**	E-mail address:
**Publication type**<br><br>*(e.g. full report, abstract, letter)*	Full report
**Study funding source**<br><br>*(including role of funders)*	None stated
**Possible conflicts of interest**<br><br>*(for study authors)*	None stated
**Notes:**	

Inclusion

Study Characteristics	Review Inclusion Criteria<br><br>*(Insert inclusion criteria for each characteristic as defined in the Protocol)*	Insert<br><br>Yes where appropriate	Location in text<br><br>*(pg & ¶/fig/table)*
**Type of study**	Non-randomised trial: Cohort		
	Non-randomised: cross sectional survey		
	Non-randomised: Case- Control study		
	Non-randomised survey		
	Non-randomised: Pre and Post with no control (pre phase only)		
	Randomised Trial (pre intervention phase)		
	Quasi-experimental trial with or without randomisation (pre intervention phase only)		
	Other design (specify):		
**Participants**	COPD patients irrespective of severity of disease living at home using a smartphone app self-management programme.		
**Concept**	The concept of interest is the development of a COPD smartphone app delivering a self-management programme, in terms of design, usability, acceptability and effect on clinical health outcomes.		
**Types of outcome measures**	Hospitalisations<br><br>User Engagement<br><br>Physical Activity<br><br>Quality of life<br><br>Self-efficacy		
**Decision: (include or exclude)**	Include		

**Study Characteristics**	**Review Inclusion Criteria**<br><br>*(Insert inclusion criteria for each characteristic as defined in the Protocol)*	**Insert**<br><br>**Yes where appropriate**	**Location in text**<br><br>*(pg & ¶/fig/table)*
**Reason for exclusion**			
**Notes**			
